# MANF Inhibits *α*-Synuclein Accumulation through Activation of Autophagic Pathways

**DOI:** 10.1155/2022/7925686

**Published:** 2022-07-08

**Authors:** Jing-Xing Zhang, Wei-Fang Tong, Ming Jiang, Kai-Ge Zhou, Xue-rui Xiang, Yi-jing He, Zhuo-yu Zhang, Qiang Guan, Ling-Jing Jin

**Affiliations:** ^1^Department of Neurology, Tongji Hospital, School of Medicine, Tongji University, Shanghai 200092, China; ^2^Biomedical Research Center, Tongji University Suzhou Institute, Jiangsu 215101, China; ^3^Department of Neurology and Neurological Rehabilitation, Shanghai Yangzhi Rehabilitation Hospital, School of Medicine, Tongji University, Shanghai 200092, China; ^4^Neurotoxin Research Center of Key Laboratory of Spine and Spinal Cord Injury Repair and Regeneration of Ministry of Education, Tongji Hospital, School of Medicine, Tongji University, Shanghai 200092, China; ^5^Shanghai Clinical Research Center for Aging and Medicine, Shanghai 200040, China

## Abstract

Progressive accumulation of misfolded SNCA/*α*-synuclein is key to the pathology of Parkinson's disease (PD). Drugs aiming at degrading SNCA may be an efficient therapeutic strategy for PD. Our previous study showed that mesencephalic astrocyte-derived neurotrophic factor (MANF) facilitated the removal of misfolded SNCA and rescued dopaminergic (DA) neurons, but the underlying mechanisms remain unknown. In this study, we showed that AAV8-MANF relieved Parkinsonian behavior in rotenone-induced PD model and reduced SNCA accumulation in the substantia nigra. By establishing wildtype (WT) SNCA overexpression cellular model, we found that chaperone-mediated-autophagy (CMA) and macroautophagy were both participated in MANF-mediated degradation of SNCA^WT^. Nuclear factor erythroid 2-related factor (Nrf2) was activated to stimulating macroautophagy activity when CMA pathway was impaired. Using A53T mutant SNCA overexpression cellular model to mimic CMA dysfunction situation, we concluded that macroautophagy rather than CMA was responsible to the degradation of SNCA^A53T^, and this degradation was mediated by Nrf2 activation. Hence, our findings suggested that MANF has potential therapeutic value for PD. Nrf2 and its role in MANF-mediated degradation may provide new sights that target degradation pathways to counteract SNCA pathology in PD.

## 1. Introduction

Parkinson's disease (PD) is a neurodegenerative disease characterized by abnormal deposits of SNCA/*α*-synuclein aggregates and progressive loss of dopaminergic neurons in the substantia nigra [1, 2]. The abnormal accumulation of SNCA could induce neurodegeneration through disrupting of axonal transport, as well as impairing mitochondrial, lysosomal, proteasomal, and endoplasmic reticulum (ER) functions [3]. SNCA exists in a dynamic equilibrium among different conformations and oligomers [4], and the propensity for its aggregation may be reversed by reduction in monomeric SNCA levels which results in disaggregation of soluble oligomers [5]. Considering accumulation and propagation of misfolded SNCA in the brain is integral to the disease pathogenesis, drugs, or herbs aiming at promoting SNCA degradation may work as an efficient therapeutic strategy for PD.

Mesencephalic astrocyte-derived neurotrophic factor (MANF) has been confirmed to possess a more specific neuroprotection for dopaminergic neurons compared to other neurotrophic factors such as glial cell line-derived neurotrophic factor (GDNF) and brain-derived neurotrophic factor (BDNF) [6]. Knocking out the homologous gene of MANF in Drosophila leading to an abnormal development of dopaminergic (DA) neurons is suggesting a key role of MANF on the development and functional maintenance of DA system [7]. Our previous studies also demonstrated that MANF inhibited the loss of DA neurons in PD rats, mice, and transgenic nematode models and improve the motor function of model animals [8–12]. Considering the vital role of SNCA accumulation in the neurodegeneration of PD, we established an A53T mutant *α*-synuclein nematode model and verified that MANF could regulate the expression of autophagy-related genes, inhibited the accumulation of SNCA^A53T^, and subsequently promote the survival of DA neurons [11]. However, the specific mechanism that triggering SNCA clearance by MANF remains unknown.

Autophagy lysosome pathway (ALP) system plays a vital role in the degradation of SNCA [13]. Inhibiting ALP activity exacerbates the abnormal aggregation of SNCA [14]. ALP includes chaperone-mediated autophagy (CMA), macroautophagy, and microautophagy. Among them, CMA and macroautophagy have been verified to be associated with the clearance of SNCA [13]. CMA is a selective protein degradation process in which cytosolic proteins bearing a KFERQ motif are recognized by Hsc70, then delivered to lysosomal associated membrane protein 2a (Lamp-2A), and are eventually transported into the lysosomal lumen for degradation [15]. In macroautophagy, portions of the cytoplasm, including protein aggregates, are sequestered inside a double membrane structure which, in turn, can fuse directly with the lysosome [16]. Previous study revealed that MANF gene mutation in Drosophila inhibited the gene expression of lysosome-associated membrane protein (LAMPs), decreased the expression of proton pump V-ATPase genes, and downregulated the lysosomal hydrolysis activity, which eventually disrupted protein degradation process [17]. Thus, we assumed that the degradation of MANF on SNCA may be mediated by CMA and/or macroautophagy pathway.

In the present study, we used the rotenone-induced PD mice model to investigate the effects of MANF on Parkinsonian behavior and SNCA pathology. Meanwhile, Lenti-X™ Tet-On® 3G Inducible Expression System and SH-SY5Y cells were used to realize doxycycline (Dox) induced expression of SNCA^WT^ and SNCA^A53T^. We characterized the role of CMA and macroautophagy pathway in MANF-mediated degradation of SNCA and investigated the potential interaction between CMA and macroautophagy activation.

## 2. Results

### 2.1. AAV8-MANF Relieves Parkinsonian Behavior and Alleviated the Accumulation of SNCA in the Substantia Nigra

**Figure 1:**
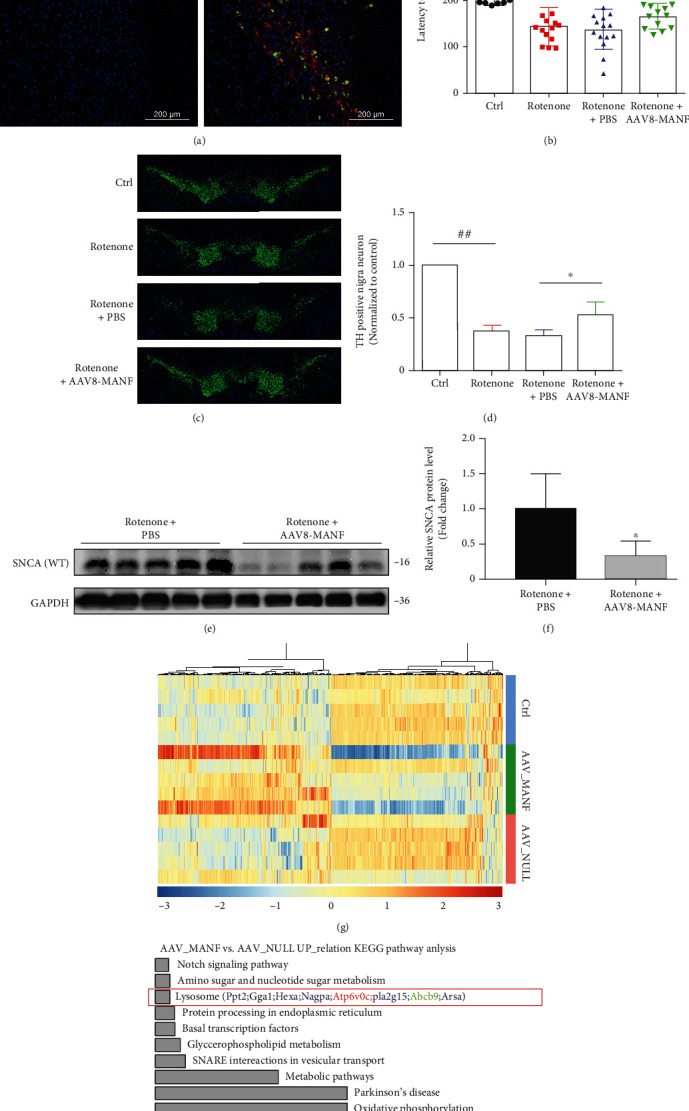
The protective effect of AAV8-MANF on PD model and its role on the expression of lysosome-related genes in substantia nigra. (a) AAV8-MANF (with His tag) was injected into the substantia nigra of rat brain. The expression of MANF in DA neurons was detected by double immunofluorescence staining using anti-His tag antibody and anti-TH antibody. Scale bar is 200 *μ*m. (b) The motor function of AAV8-MANF on rotenone-induced PD mouse models was evaluated by rotarod analysis. Ctrl group: *n* = 14; rotenone group: *n* = 14; rotenone + PBS group: *n* = 15; rotenone + AAV8 − MANF group: *n* = 13. (c, d) The effect of AAV8-MANF on rotenone-induced DA neuron degeneration was determined by TH staining. Scale bar is 500 *μ*m. (e, f) The level of SNCA in substantia nigra was detected using western blot analysis. Rotenone + PBS group and rotenone + AAV8 − MANF group: *n* = 5. (g, h) RNA-sequence analysis of gene expression in substantia nigra followed by AAV8-MANF injection. (e) Heatmap analysis. (f) KEGG pathway analysis. Data were expressed as mean ± SD from three independent experiments. ^#^*P* < 0.05 and ^##^*P* < 0.01 vs. Ctrl group; ^∗^*P* < 0.05 vs. rotenone + PBS-treated group.

AAV8-MANF (with His tag) was established to achieve the continuous supply of MANF. As shown in Figure 1(a), AAV8-MANF injected into substantia nigra could effectively infect dopaminergic neurons and express MANF protein. AAV8-MANF was injected into the bilateral substantia nigra 1 week before rotenone (3 mg/kg) was subcutaneously injected for 5 weeks. Rotarod analysis showed that AAV8-MANF significantly ameliorated motor impairments of PD mouse (Figure 1(b)). TH staining indicated that AAV8-MANF inhibited rotenone-induced DA neuron degeneration (Figures 1(c) and 1(d)). Afterward, as indicated in Figures 1(e) and 1(f), the increased accumulation of SNCA in substantia nigra was decreased following AAV8-MANF treatment. RNA-seq sequencing revealed that AAV8-MANF could promote the expression of genes include membrane protein genes (Abcb9), hydrolase genes (Ppt2, Hexa, Arsa, Nagpa, and Pla2g15), and proton pump V-ATPase gene (Atp6v0c) related to the lysosomal pathway, indicating an involved of ALP in MANF-mediated SNCA degradation (Figures 1(g) and 1(h)).

### 2.2. MANF Inhibits the Accumulation of SNCA^WT^ in PD Cellular Model

**Figure 2:**
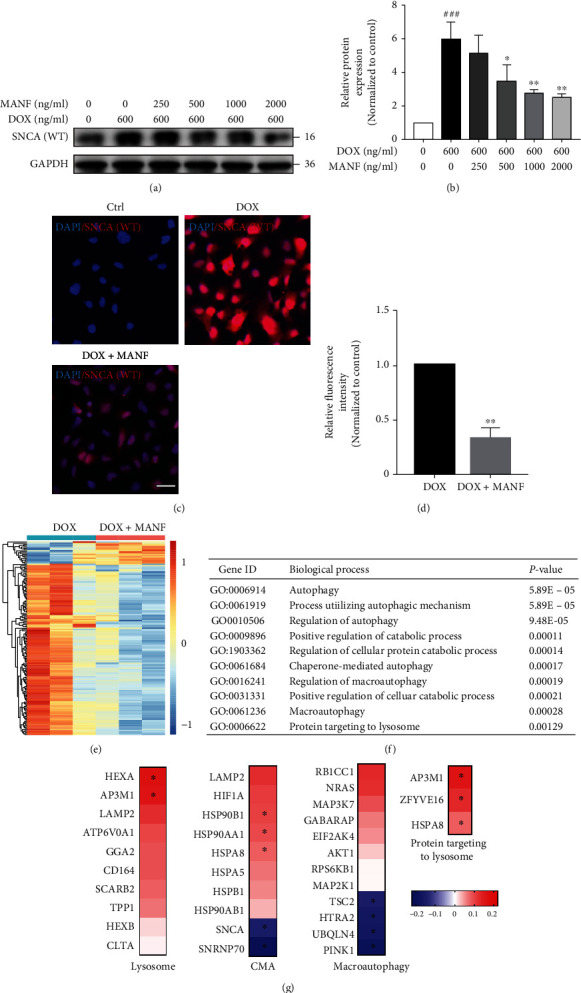
Effects of MANF on the degradation of SNCA^WT^ in PD cellular model. (a, b) SNCA^WT^ SH-SY5Y cells were treated with MANF (0, 250, 500, 1000, or 2000 ng/ml) and Dox (600 ng/ml) for 24 h, and cell lysates were immunoblotted by anti-SNCA antibody. (c, d) SNCA^WT^ SH-SY5Y cells were treated with MANF (500 ng/ml) and Dox (600 ng/ml) for 24 h, and the level of SNCA^WT^ was detected by immunofluorescence staining. Scale bar is 50 *μ*m. (e)–(g) SNCA^WT^ H-SY5Y cells were treated with MANF (500 ng/ml) and Dox (600 ng/ml) for 24 h, followed by RNA-sequence analysis. (e) Heatmap analysis. (f) KEGG pathway analysis. (g) Target genes involved in lysosomal-related degradation pathway. Data were expressed as mean ± SD from three independent experiments. ^###^*P* < 0.001 vs. control group; ^∗^*P* < 0.05 and ^∗∗^*P* < 0.01 vs. Dox-treated group.

Lenti-X™ Tet-On® 3G inducible expression system and SH-SY5Y cells were used to realize Dox-induced expression of SNCA^WT^ (Figure S1 A-C). MANF treatment alone did not show any obvious effect on the viability of SNCA^WT^ SH-SY5Y cells for 48 h (Figure S3). Whereas MANF treatment for 24 h significantly reduced the accumulation of SNCA^WT^ in a concentration dependent manner (Figures 2(a)–2(d)). RNA-seq sequencing and subsequently BP analysis showed that the expression of genes associated with “Macroautophagy,” “Chaperone-mediated autophagy (CMA),” “Protein targeting to lysosome,” and other genes involved in ALP process was changed after MANF treatment for 24 h (Figures 2(e)–2(g)).

### 2.3. Involvement of CMA in MANF-Mediated Degradation of SNCA^WT^

**Figure 3:**
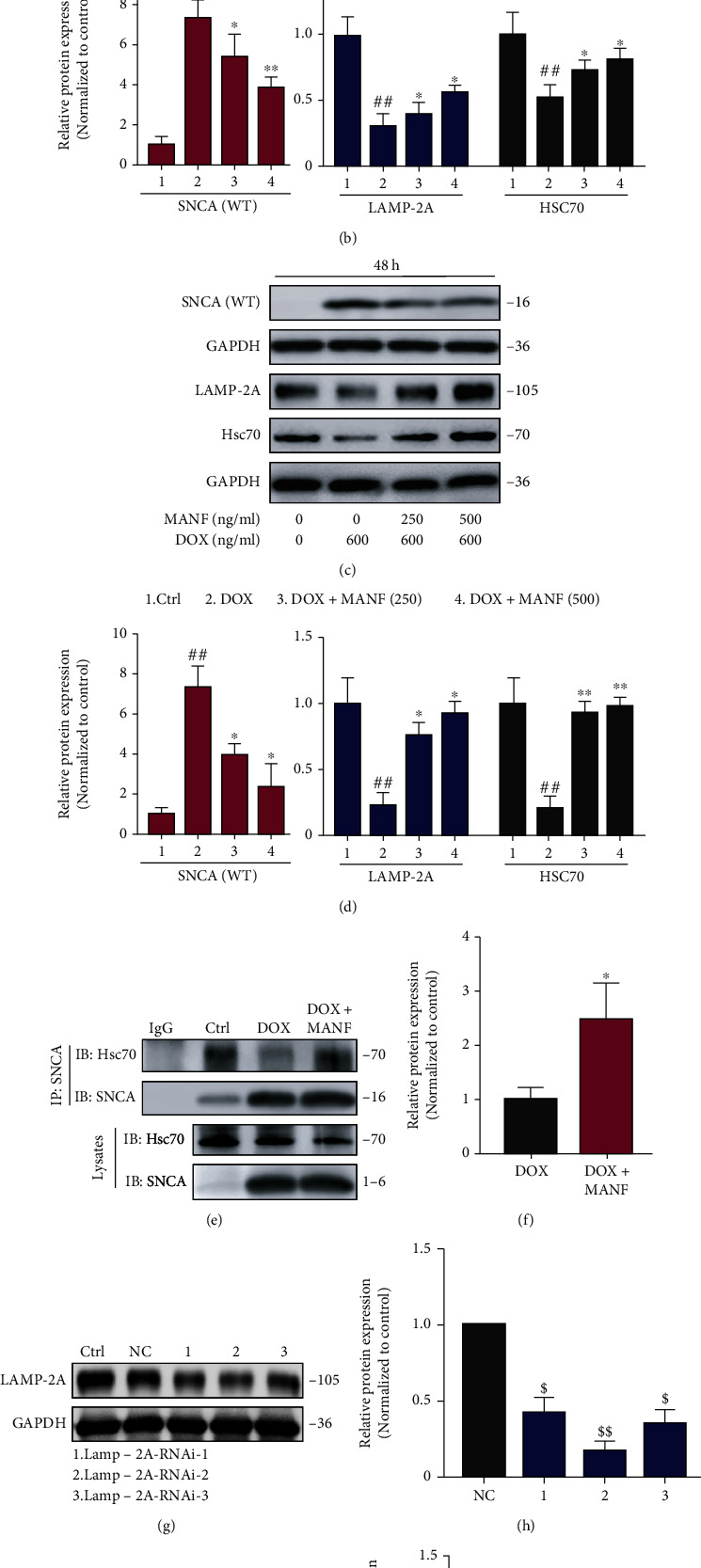
MANF inhibited the accumulation of SNCA^WT^ in PD cellular model by CMA activation. (a)–(d) Effects of MANF on the levels of SNCA^WT^, Lamp-2A, and Hsc70. SNCA^WT^ SH-SY5Y cells were treated with MANF (250, 500 ng/ml) and Dox (600 ng/ml) for 24 h (a, b) or 48 h (c, d), respectively; then, the protein levels were detected by western blot analysis. (e, f) Effects of MANF on the interaction between SNCA^WT^ and Hsc70. SNCA^WT^ SH-SY5Y cells were treated with MANF (500 ng/ml) and Dox (600 ng/ml) for 24 h. Cell lysates were immunoprecipitated with anti-SNCA, and the precipitated proteins were analyzed by immunoblotting with anti-Hsc70. (g, h) SNCA^WT^ SH-SY5Y cells were treated with Lamp-2A RNAi 1-3 for 48 h. The level of LAMP-2A was detected by western blot analysis. (i, j) Lamp-2A knocked down partly abolished MANF-induced SNCA^WT^ clearance. SNCA^WT^ SH-SY5Y cells were treated with Lamp-2A RNAi 2 for 24 h, followed by the incubation of MANF (500 ng/ml) and Dox (600 ng/ml) for another 24 h. The levels of LAMP-2A and SNCA^WT^ were detected by western blot analysis. Data were expressed as mean ± SD from three independent experiments. ^##^*P* < 0.01 vs. control group; ^$^*P* < 0.05 vs. NC group; ^∗^*P* < 0.05 and ^∗∗^*P* < 0.01 vs. Dox-treated group; ^&^*P* < 0.05 vs. combined treatment with negative control Lamp-2A RNAi plasmid, MANF, and Dox group.

To identify the role of CMA in MANF-induced degradation, the expression of SNCA^WT^, LAMP-2A, and Hsc70 was detected using western blotting. As shown in Figures 3(a) and 3(b), MANF markedly attenuated Dox-induced accumulation of SNCA^WT^. Meanwhile, the expression of LAMP-2A and Hsc70 was significantly increased after MANF treatment (Figures 3(a)–3(d)), indicating the activation of CMA. In addition, Co-IP assay revealed that the combination of SNCA^WT^ and Hsc70 was enhanced, suggesting a convenient transportation for SNCA^WT^ to bind LAMP-2A on lysosome (Figures 3(e) and 3(f)). However, MANF-mediated degradation of SNCA^WT^ was almost completely reversed when CMA pathway was blocked using RNAi-based approach (Figures 3(g)–3(j)). Together, these results point to the association of MANF-mediated CMA activation in reducing SNCA^WT^ accumulation.

### 2.4. Macroautophagy Was Involved in MANF-Mediated Degradation of SNCA^WT^

**Figure 4:**
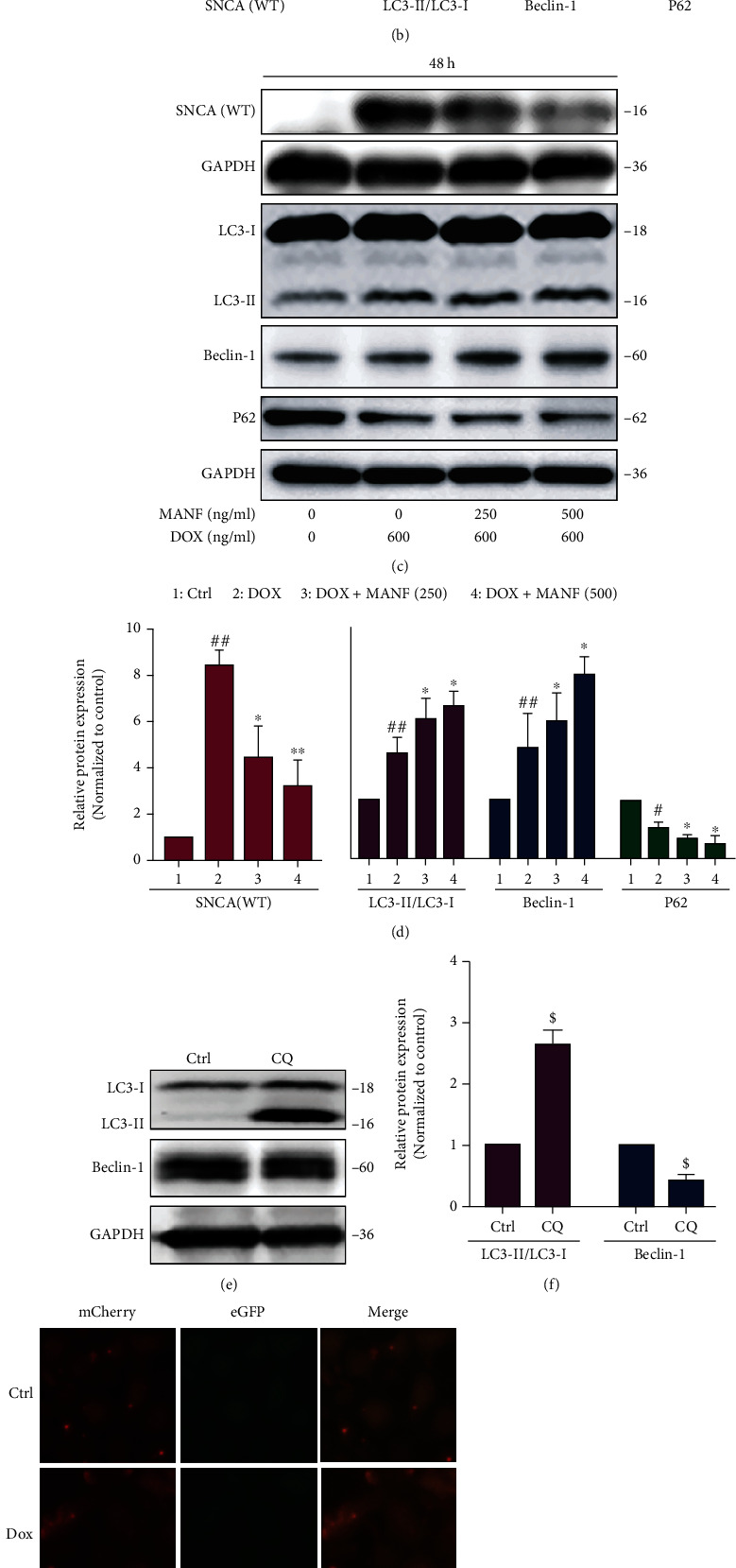
MANF inhibits the accumulation of SNCA^WT^ in PD cellular model by macroautophagy activation. (a)–(d) Effects of MANF on the levels of SNCA^WT^ and macroautophagic-related protein expression. SNCA^WT^ SH-SY5Y cells were treated with MANF (250 and 500 ng/ml) and Dox (600 ng/ml) for 24 h (a, b) or 48 h (c, d), respectively; then, the protein levels of SNCA, LC3-I/II, Beclin-1, and P62 were detected by western blot analysis. (e, f) SNCA^WT^ SH-SY5Y cells were treated with autophagy inhibition CQ (10 *μ*M) for 48 h, and cell lysates were immunoblotted by anti-LC3 and anti-Beclin-1 antibodies. (g, h) After SNCA^WT^ SH-SY5Y cells were treated with Lenti-mCherry-eGFP-LC3B plasmid for 48 h, MANF (500 ng/ml), Dox (600 ng/ml), and CQ (10 *μ*M) were added, respectively, for another 48 h. The fluorescence of mCherry and eGFP was observed using fluorescence microscopy and quantified by Image Pro Plus software. Scale bar is 50 *μ*m. (i, j) SNCA^WT^ SH-SY5Y cells were treated with MANF (500 ng/ml), Dox (600 ng/ml), and CQ (10 *μ*M) for 48 h. The protein level of SNCA^WT^ was detected by western blot analysis. Data were expressed as mean ± SD from three independent experiments. ^#^*P* < 0.05, ^##^*P* < 0.01, and ^$^*P* < 0.05 vs. control group; ^∗^*P* < 0.05 and ^∗∗^*P* < 0.01 vs. Dox-treated group; ^&^*P* < 0.05 vs. combined treatment with MANF and Dox group.

Macroautophagy was reported to be activated to promote SNCA clearance when CMA system is impaired caused by SNCA overaccumulation [13]. Here, we tested whether MANF-mediated degradation of SNCA^WT^ was modulated by macroautophagy. As shown in Figures 4(a) and 4(b), when cells were incubated with Dox and MANF for 24 h, the content of SNCA^WT^ was significantly reduced, but the expression of macroautophagy markers such as light chain 3 (LC3), Beclin-1, and P62 were not changed, indicating that macroautophagy system was not activated in the early stagy. However, when cells were incubated with Dox and MANF for 48 h, the conversion of LC3-I/II and the expression of Beclin-1 were increased, and simultaneously, the expression of P62 was decreased by MANF treatment, suggesting macroautophagy was activated during SNCA accumulation (Figures 4(c) and 4(d)). By using Lenti-mCherry-eGFP-LC3B plasmid and macroautophagic inhibitor CQ to detect autophagic flux, we found that MANF could partly inhibit the decreased ratio of mCherry/eGFP fluorescence intensity caused by CQ, indicating an activation of macroautophagy (Figures 4(e)–4(h)). More importantly, the clearance of MANF on SNCA^WT^ was partially abolished by the treatment of macroautophagic inhibitor CQ (Figures 4(i)–4(j)). These findings indicated that, besides CMA, macroautophagy system was also involved in MANF-mediated degradation on SNCA^WT^.

### 2.5. Nrf2 Was Involved in the Early Activation of Macroautophagy by MANF when CMA System Was Blocked

**Figure 5:**
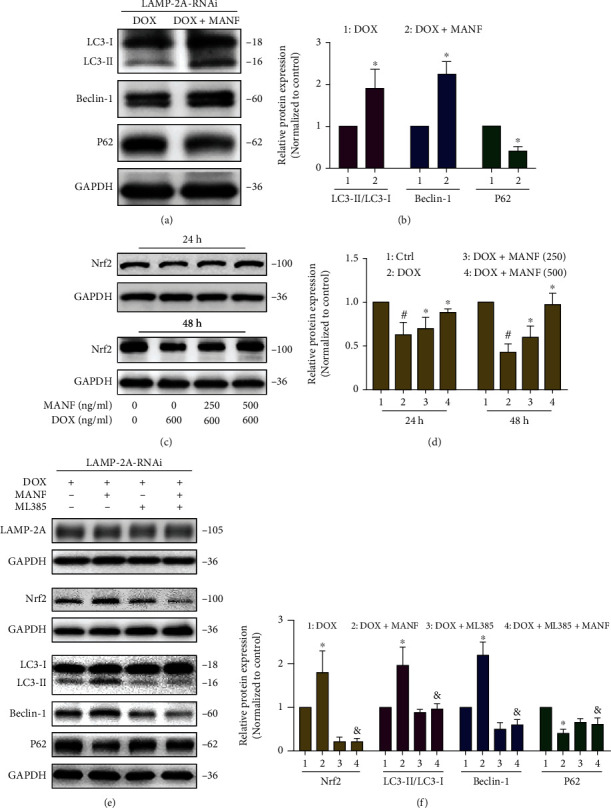
Effects of Nrf2 on the early activation of macroautophagy by MANF. (a, b) SNCA^WT^ SH-SY5Y cells were treated with Lamp-2A-RNAi 2 plasmid for 24 h, followed by incubation with MANF (500 ng/ml) and Dox (600 ng/ml) for another 24 h. The levels of LC3, Beclin-1, and P62 were detected by western blot analysis. (c, d) SNCA^WT^ SH-SY5Y cells were treated with MANF (500 ng/ml) and Dox (600 ng/ml) for another 24 h or 48 h, respectively; cell lysates were immunoblotted by anti-Nrf2 antibody. (e, f) SNCA^WT^ SH-SY5Y cells were treated with Lamp-2A-RNAi 2 plasmid for 24 h, followed by incubation with MANF (500 ng/ml), ML385 (5 *μ*M), and Dox (600 ng/ml) for another 24 h. The levels of LAMP-2A, Nrf2, LC3, Beclin-1, and P62 were detected by western blot analysis. Data were expressed as mean ± SD from three independent experiments. ^#^*P* < 0.05 vs. control group; ^∗^*P* < 0.05 vs. Dox-treated group; ^&^*P* < 0.05 vs. combined treatment with MANF and Dox group.

To evaluate the crosstalk or compensatory interaction between CMA and macroautophagy, CMA activation was suppressed by RNAi-mediated LAMP-2A inhibition. As shown in Figures 5(a) and 5(b), the conversion of LC3-I/II and the expression of Beclin-1 were increased, while the expression of P62 was decreased when CMA system was blocked, indicating a beforehand activation of macroautophagy by MANF.

Both CMA and macroautophagy pathway could be regulated by Nrf2 [18, 19]. Upregulating Nrf2 reduced the abnormal accumulation of SNCA by promoting CMA and macroautophagy activity [20]. Besides, when macroautophagy was blocked, P62 could activate Nrf2 by binding to keap1 [21] that may trigger other degradation pathway, such as CMA. We reported that MANF increased Nrf2 expression and promoted its nuclear translocation to exerting antiapoptotic effects [9]. Hence, we speculated that Nrf2 may be involved in the crosstalk between CMA and macroautophagy on MANF-induced clearance of SNCA^WT^. As shown in Figures 5(c) and 5(d), MANF promoted the expression of Nrf2. ML385, a widely used inhibitor of Nrf2, significantly reversed the MANF-induced activation of macroautophagy in CMA impaired condition (Figures 5(e) and 5(f)).

**Figure 6:**
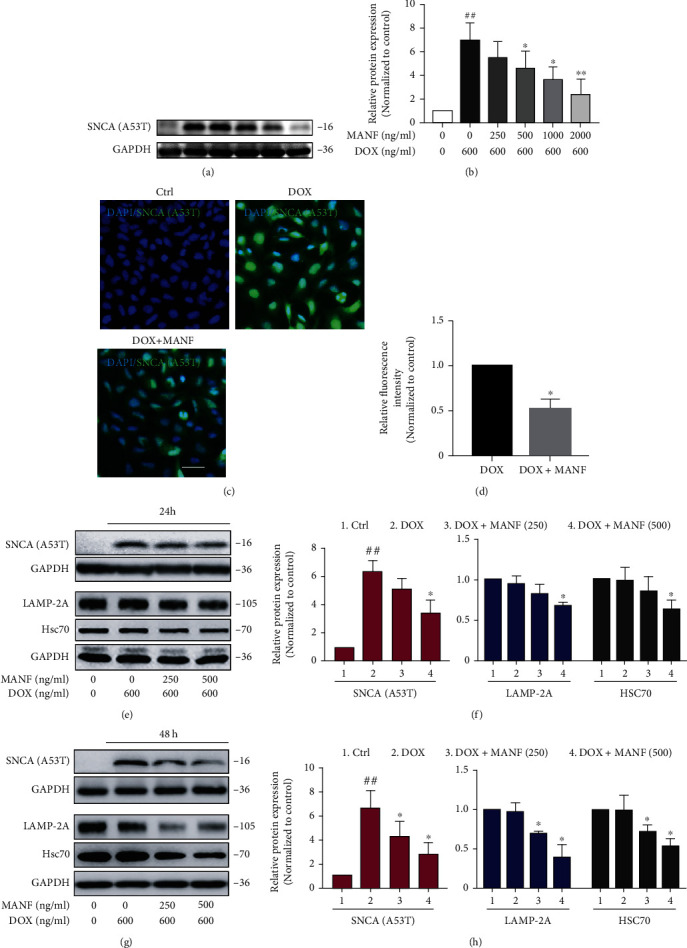
MANF inhibited the accumulation of SNCA^A53T^ in PD cellular model. (a, b) SNCA^A53T^ SH-SY5Y cells were treated with MANF (0, 250, 500, 1000, or 2000 ng/ml) and Dox (600 ng/ml) for 24 h, and cell lysates were immunoblotted by anti-SNCA antibody. (c, d) SNCA^A53T^ SH-SY5Y cells were treated with MANF (500 ng/ml) and Dox (600 ng/ml) for 24 h, and the level of SNCA was detected by immunofluorescence staining. Scale bar is 50 *μ*m. (e)–(h) Effects of MANF on the levels of SNCA, Lamp-2A, and Hsc70. SNCA^A53T^ SH-SY5Y cells were treated with MANF (250 and 500 ng/ml) and Dox (600 ng/ml) for 24 h (a, b) or 48 h (c, d), respectively; then, the protein levels were detected by western blot analysis. Data were expressed as mean ± SD from three independent experiments. ^##^*P* < 0.01 vs. control group; ^∗^*P* < 0.05, ^∗∗^*P* < 0.01 vs. Dox-treated group.

**Figure 7:**
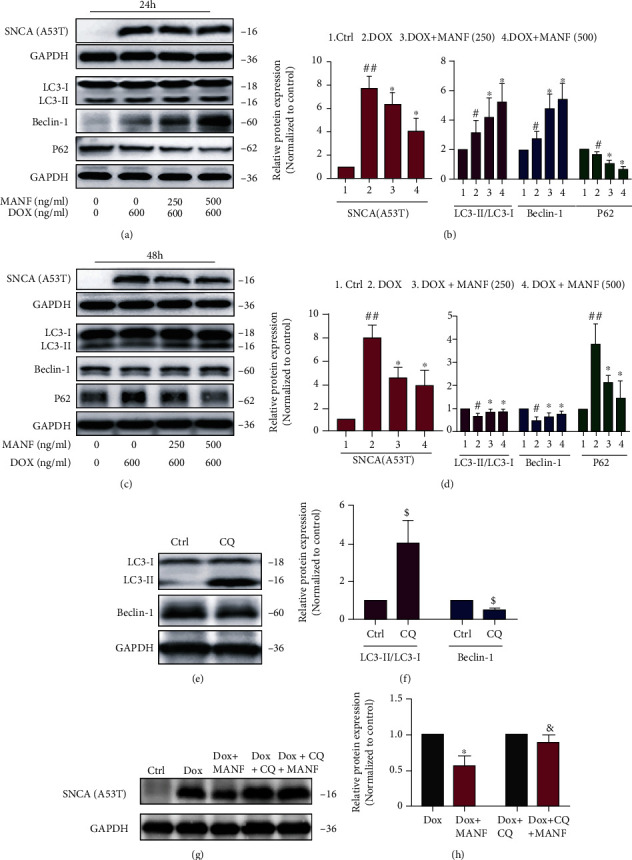
MANF inhibits the accumulation of SNCA^A53T^ in PD cellular model by activating macroautophagy. (a)–(d) Effects of MANF on the levels of SNCA^A53T^ and macroautophagic-related protein expression. SNCA^A53T^ SH-SY5Y cells were treated with MANF (250 and 500 ng/ml) and Dox (600 ng/ml) for 24 h (a, b) or 48 h (c, d), respectively; then, the protein levels of SNCA^A53T^, LC3-I/II, Beclin-1, and P62 were detected by western blot analysis. (e, f) SNCA^A53T^ SH-SY5Y cells were treated with autophagy inhibition CQ (10 *μ*M) for 24 h, and cell lysates were immunoblotted by anti-LC3 and anti-Beclin-1 antibodies. (g, h) SNCA^A53T^ SH-SY5Y cells were treated with MANF (500 ng/ml), Dox (600 ng/ml), and CQ (10 *μ*M) for 48 h, and the protein level of SNCA^A53T^ was detected by western blot analysis. Data were expressed as mean ± SD from three independent experiments. ^#^*P* < 0.01, ^##^*P* < 0.01, and ^$^*P* < 0.05 vs. control group; ^∗^*P* < 0.05 vs. Dox-treated group; ^&^*P* < 0.05 vs. combined treatment with MANF and Dox group.

**Figure 8:**
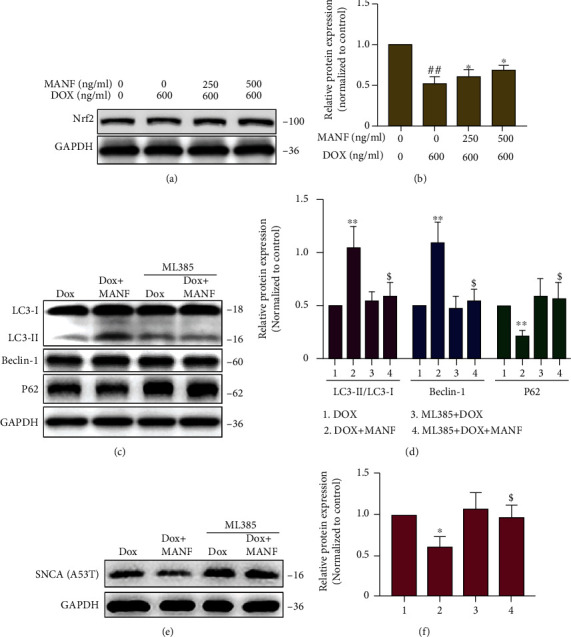
Effects of Nrf2 on the activation of macroautophagy by MANF in SNCA^A53T^ SH-SY5Y cells. (a, b) SNCA^A53T^ SH-SY5Y cells were treated with MANF (500 ng/ml) and Dox (600 ng/ml) for 24 h, and cell lysates were immunoblotted by anti-Nrf2 antibody. (c)–(f) SNCA^A53T^ SH-SY5Y cells were treated with MANF (500 ng/ml), Dox (600 ng/ml), and ML385 (5 *μ*M) for 24 h, and the protein levels of LC3-I/II, Beclin-1, P62, and SNCA^A53T^ were detected by western blot analysis. Data were expressed as mean ± SD from three independent experiments. ^##^*P* < 0.01 vs. control group; ^∗^*P* < 0.05 vs. Dox-treated group; ^$^*P* < 0.05 vs. combined treatment with MANF and Dox group.

Cuervo et.al reported that SNCA^A53T^ could block CMA due to the strongly bound to LAMP-2A and disrupt the degradation of substrate proteins [22]. Hence, SNCA^A53T^ overexpression cellular model was used to mimic CMA dysfunction situation, and Nrf2 mediated activation of macroautophagy was investigated. Lenti-X™ Tet-On® 3G inducible expression system and SH-SY5Y cells were used to realize Dox induced expression of SNCA^A53T^ (Figure S2A-C). We showed that MANF treatment significantly reduced the accumulation of SNC^A53T^ in a concentration dependent manner (Figures 6(a)–6(d)). As expected, CMA was not activated in MANF-mediated clearance of SNCA^A53T^ due to impaired CMA system (Figures 6(e)–6(h)), while macroautophagy was confirmed to be participated in the degradation of SNCA^A53T^ by MANF (Figures 7(a)–7(h)). Noticeably, Nrf2 inhibitor ML385 could partially abrogate MANF-mediated activation of macroautophagy and the subsequent clearance of SNCA^A53T^ (Figures 8(a)–8(f)). Accordingly, these findings suggest that Nrf2 was involved in the early activation of macroautophagy by MANF when CMA system was impaired.

## 3. Discussion

SNCA plays a central role in the aetiology and pathophysiology of PD [23]. Drugs or herbs that own SNCA clearance properties may be potential candidates for PD treatment. MANF, as a newly identified neurotrophic factor, has been confirmed to possess neuroprotective effects on PD [24]. Our previous study further demonstrated that MANF could activate autophagy-related genes and alleviate the aggregation of SNCA^A53T11^, but the exact mechanisms remain unknown. In the current study, we firstly confirmed that CMA and macroautophagy, the two main subtypes of ALP, were both involved in MANF-induced degradation of SNCA^WT^. In addition, we found that MANF could activate macroautophagy in a Nrf2-dependent manner when CMA system was impaired.

CMA is a selective degradative process for cytosolic proteins that contributes to the maintenance of proteostasis [25]. Reduced levels of CMA markers have been observed in postmortem brain samples from PD patients [26], indicating that CMA dysfunction was associated with the pathogenesis of PD. In addition, SNCA, the neuropathological hallmark, also contains KFERQ sequence, which was verified as a natural substrate of CMA [26]. Increasing evidence confirmed that CMA represents a major pathway for SNCA^WT^ degradation [27]. CMA inhibition was accompanied by formation of detergent-insoluble or high molecular-weight (HMW) oligomeric SNCA conformations, leading to dopaminergic neurodegeneration and parkinsonian behavior [28]. Our observations revealed that MANF could upregulate the expression of HSC70 and LAMP-2A, the main components of CMA, and promote the combination between HSC70 and SNCA^WT^, indicating an activation of CMA system, which eventually contributed to the degradation of SNCA^WT^.

Only SNCA monomers and dimers, but not oligomers, could be degraded by CMA [29]. Excess levels and/or abnormal aggregated of SNCA impaired CMA, and then, macroautophagy system will be activated to participate in the clearance of oligomer SNCA [30]. Using a Dox-induced cellular model of SNCA^WT^, we also found that macroautophagy system would be activated by MANF due to the continuously accumulation of SNCA^WT^, which suggested that CMA could participate in the degradation of SNCA^WT^ in the early stagy and macroautophagy system may act as a subsequent compensation mechanism. Interestingly, we revealed that when CMA was blocked by using a LAMP-2A targeting siRNA-based approach, or using Dox-induced SNCA^A53T^ overexpression cell model to mimic CMA dysfunctional condition, macroautophagy could be early activated by MANF to eliminate the accumulation of SNCA^WT^. These results indicated that MANF could promote the degradation of SNCA^WT^from SNCA accumulation to aggregation. Meanwhile, even when CMA was blocked due to uncontrolled accumulation of SNCA, MANF still could opportunely activate macroautophagy to further degrade SNCA, which is important for inhibiting SNCA-induced neurotoxicity.

Nrf2, as a leucine zipper transcription factor, controls the basal and stress-inducible expression of over 250 genes [31]. A majority of these genes are involved in the different phases of the macroautophagy process, from cargo recognition to autolysosome clearance [32–36]. Nrf2 can also activate macroautophagy through the Nrf2-p62-keap1 feedback loop [19]. Besides, Nrf2 could recognize and bind to the ARE functional region in the LAMP-2A gene, thereby regulating the expression of LAMP-2A and eventually upregulating CMA activity [18]. Thus, the Nrf2 pathway could serve as an upstream signal to regulate the activities of both CMA and macroautophagy [18, 20]. Genetic increase of astrocyte-specific Nrf2 could attenuate the functional deficiency of macroautophagy and CMA, which promoted the degradation of SNCA^A53T^ in SNCA^A53T^ mouse model [20]. Hence, Nrf2 might act as a regulatory node in the proteolytic network represented by macroautophagy and CMA in PD and may serve as a link whereby one reacts in a compensatory manner to the loss of activity in the other [19–21]. In this study, we found that MANF upregulated Nrf2 expression while stimulating CMA or macroautophagy system. Blockage of Nrf2 using ML385 (a specific inhibitor) could almost completely counteract the early activation of macroautophagy induced by MANF. These results revealed that Nrf2 was involved in the functional compensatory between CMA and macroautophagy by mediating macroautophagy activation in CMA dysfunction situation.

## 4. Conclusions

Our results demonstrated that CMA and macroautophagy system are involved in MANF-induced degradation of SNCA^WT^. Meanwhile, Nrf2 was confirmed to be a regulator to activate macroautophagy in CMA dysfunctional situation. There might be some limitations in the present study. (I) Ubiquitin-proteasome system (ULP) is also involved in the regulation of SNCA. Monoubiquitinated SNCA is targeted for degradation by the proteasomal system [37]. Whether MANF-mediated degradation of SNCA is associated with ULP needs further exploration. (II) The exact mechanisms of Nrf2 on MANF-induced regulation of CMA and macroautophagy still need to be clarified. (III) Considering the multiple mechanisms involved in the activation of CMA and macroautophagy, besides Nrf2, other signaling pathways need to be further figured out. Despite some limitations, the findings from our investigation indicated MANF may be a candidate for the treatment of PD. Meanwhile, Nrf2 and its role in MANF-mediated degradation may provide new therapeutic strategies that target degradation pathways to counteract SNCA pathology in PD.

## 5. Materials and Methods

### 5.1. Animals and Treatment

In previous studies, adult male rats and mice have been widely used in the study of PD [38–41]. In this study, adult male Sprague-Dawley rats (used for detecting the expression of AAV8-MANF and RNA-seq) and male C57/BL6 mice (used for exploring the protection of AAV8-MANF on Rotenone-induced PD models) were obtained from Shanghai SLAC Laboratory Animal Co., Ltd. (Shanghai, China) and used following the National Institutes of Health Guide for the Care and Use of Laboratory Animals and approved by the Animal Use and Care Committee of Shanghai Tongji Hospital. Rotenone (Sigma, St. Louis, USA) was dissolved in dimethyl sulfoxide (Sigma, St. Louis, USA). C57/BL6 mouse received subcutaneous injections of Rotenone (3 mg/kg) for 5 weeks to establish PD model. Mice that received formulation buffer were regarded as control.

### 5.2. Cell Culture and Treatment

SNCA^WT^ SH-SY5Y cells and SNCA^A53T^ SH-SY5Y cells were established using Lenti-X™ Tet-On® 3G inducible expression system and SH-SY5Y cells according to user manual. Doxycycline (Dox; Clontech Laboratories, CA, USA) was used to induce the expression of SNCA^WT^ and SNCA^A53T^. Cells were routinely grown in Dulbecco's Modified Eagle's Medium (DMEM; Invitrogen, Carlsbad, CA, USA) supplemented with 10% tetracycline-free fetal bovine serum (Clontech Laboratories, CA, USA) and cultured at 37°C under humidified 5% CO2 atmosphere. When cells were subcultured once attaining 70-80% confluency, MANF (PeproTech, State of NJ, USA) and Dox were added for 24 or 48 h, respectively.

### 5.3. Construction of AAV8-MANF-His and AAV8-NULL

The coding regions of the MANF with His tagged to its C-terminal were synthesized by Genewiz bio (South Plainfield, NJ, USA). The synthesized genes were digested with the relevant restriction enzymes, and DNA fragments were ligated into the Stratagene CMV AAV expression vector yielding the vector pAAV-MANF-His was cloned into the Stratagene CMV AAV expression vector to generate the control virus. Constructs were verified by sequencing and packaged into AAV8 viral particles with the Rep2/Cap8 AAV encapsulation construct. AAV8-NULL was packaged as the negative control of AAV8-MANF-His. These two viral vectors were purified by a 2-step chromatographic step.

### 5.4. Intrastriatal Injection of AAV8-MANF-His

Anesthetized mouse received injection of AAV8-MANF-His (1 × 10^13^ v.g/ml) to the bilateral substantia nigra (SN) according to the following stereotaxic coordinates: AP, -3.0 mm; ML, -/+1.3 mm; DV, -4.7 mm; at a continuous flow rate of 0.5 *μ*l/min using standard stereotaxic surgical procedures. The injection volume was 1 *μ*l. The rats received injection of AAV8-MANF-His and AAV8-NULL into the unilateral SN according to the following stereotaxic coordinates: AP, -5.5 mm; ML, -2.2 mm; DV, -8.2 mm; at a continuous flow rate of 0.5 *μ*l/min. The injection volume was 3 *μ*l.

### 5.5. Rotarod Test

Rotarod analysis (Harvard apparatus, Holliston, MA, USA) was performed by placing the animals on an accelerating rod (4-40 r.p.m. over the course of a 5 min trial, 30 min intertrial interval). All animals were trained 3 days before rotarod test. Latency to fall was recorded for 3 days (3 trials/day). Motor test data were presented as the percentage of the mean duration on the rotarod compared to the control.

### 5.6. Bioinformatic Analysis

After AAV8-MANF was injected into the right SN of rats for 4 weeks or SNCA^WT^ SH-SY5Y cells were treated with MANF and Dox for 24 h. Total RNA of samples was extracted by RNAiso Plus (Thermo Fisher Scientific, MA, USA) following the product's descriptions for RNA-seq. RNA-seq libraries were constructed by Novogene (Beijing, China) and sequenced by Hiseq-4000 system (Illumina). Reads of RNA-seq were aligned to human reference genome (hg19) or mouse reference genome (mm10) by the spliced read aligner Hisat2 (vision 2.1.0). Then, gene abundances were estimated and normalized to fragments per kilobase of transcript per million fragments mapped (FPKM) by StringTie (version 2.2.1), and read counts of genes were summarized by HTSeq (vision 0.7.2). Differential expression analysis of genes in this study was identified by “DESeq2” package, and genes with adjusted *P* value < 0.05 were considered as differentially expressed. GO and KEGG term gene function enrichment analysis and visualization are by “Cluster Profiler” package.

### 5.7. Immunohistochemistry

Frozen section of brain containing SN or cells was fixed and blocked by blocking solution (Beyotime Biotechnology, Nantong, China) for 1 h at RT and then incubated overnight with primary antibodies (anti-His antibody from Cell Signaling Technology, Beverly, USA; anti-SNCA antibody from Abcam, MA, USA; anti-TH antibody from Millipore, Bedford, MA, USA) at 4°C. The slices or cells were washed three times in PBS and then incubation with Alexa Fluor® 488 or Alexa Fluor® 594-conjugated goat antirabbit antibodies (both from Abcam, MA, USA) for 60 min at 37°C. After the slices or cells were washed three times in PBS, the fluorescence was observed using fluorescence microscopy.

### 5.8. Western Blot

After treating under various conditions, the tissues or cells were harvested, the total proteins were extracted, and the total protein concentrations were detected by BCA Protein Assay Kit (Beyotime Biotechnology, Nantong, China). Equivalent amounts of protein of each sample were electrophoresed on SDS-polyacrylamide gels and transferred to polyvinylidene difluoride membranes (Millipore, Bedford, MA, USA). After the membranes were blocked for 1 h at RT, primary antibodies (in TBST-5% BSA) against SNCA, LAMP-2A, Hsc70, LC3, Beclin-1, P62, and Nrf2 (anti-SNCA, anti-LAMP-2A, anti-Hsc70, and anti-Nrf2 antibodies are obtained from Abcam, MA, USA; anti-LC3, anti-Beclin-1, and anti-P62 antibodies are obtained from Cell Signaling Technology, Beverly, USA) were added and incubated overnight at 4°C. After being washed 3 times in TBST, the membranes were incubated with HRP-conjugated secondary antibodies (KPL, Gaithersburg, MD, USA) for 1 h at 37°C. An ECL kit (Millipore, Bedford, MA, USA) was used to visualize membrane immunoreactivity.

### 5.9. Coimmunoprecipitation

The cell samples were lysed in lysis buffer (Beyotime Biotechnology, Nantong, China) and centrifuged for 15 min at 16,000 g. The supernatant was incubated with anti-SNCA antibody and Protein A/G–agarose (Beyotime Biotechnology, Nantong, China) overnight at 4°C. After extensive washing with lysis buffer, the bound proteins were eluted from the beads by boiling in loading buffer and were subjected to western blot analyses.

### 5.10. RNA Interference and Gene Transfection

The transfection of short hairpin RNA (ShRNA) was used to downregulate the expression of LAMP-2A (TargetSeq: GCAGCATCTACTTATTCAATT). Lenti-mCherry-eGFP-LC3B plasmid was used to determine autophagic flux. The human SNCA^WT^ SH-SY5Ycells were plated into 12-well plates at the density of 2 × 10^5^ cells/well and then transfected with LAMP-2A ShRNA or Lenti-mCherry-eGFP-LC3B plasmid (both from Genechem, Shanghai, China) using transfection reagent lipofectamine 3000 (Invitrogen, Shanghai, China), according to the manufacturer's protocol.

### 5.11. Statistical Analysis

Data visualization and analysis were performed with GraphPad Prism 8 (GraphPad Software Inc., La Jolla, CA, USA). And a one-way analysis of variance (ANOVA) followed either student's *t*-test by was used to compare each experiment. For all statistical analyses, *P* < 0.05 was considered significant.

## Data Availability

The datasets used and/or analyzed during the current study are available from the corresponding author on reasonable request.

## Abbreviations

PD: Parkinson's disease
MANF: Mesencephalic astrocyte-derived neurotrophic factor
SNCA: *α*-Synuclein
DA: Dopaminergic
WT: Wildtype
CMA: Chaperone-mediated-autophagy
Nrf2: Nuclear factor erythroid 2-related factor
ER: Endoplasmic reticulum
GDNF: Glial cell line-derived neurotrophic factor
BDNF: Brain-derived neurotrophic factor
ALP: Autophagy lysosome pathway
LAMP: Lysosome-associated membrane protein
Dox: Doxycycline.

## Supplementary Materials

Supplementary Materials Figure S1: western blot analysis and immunofluorescence staining of DOX-induced expression of WT SNCA. Figure S2: western blot and immunofluorescence analysis of DOX-induced expression of A53T SNCA. Figure S3: cell viability analysis of SNCA^WT^ SH-SY5Y cells after MANF treatment.

## References

[1] Kalia L. V., Lang A. E. (2015). Parkinson's disease. *Lancet (London, England)*.

[2] Schneider R. B., Iourinets J., Richard I. H. (2017). Parkinson's disease psychosis: presentation, diagnosis and management. *Neurodegenerative disease management.*.

[3] Miller K. M., Mercado N. M., Sortwell C. E. (2021). Synucleinopathy-associated pathogenesis in Parkinson's disease and the potential for brain-derived neurotrophic factor. *NPJ Parkinson's disease.*.

[4] Brucale M., Sandal M., Di Maio S. (2009). Pathogenic mutations shift the equilibria of alpha-synuclein single molecules towards structured conformers. *Chembiochem: a European journal of chemical biology.*.

[5] Cremades N., Cohen S. I., Deas E. (2012). Direct observation of the interconversion of normal and toxic forms of *α*-synuclein. *Cell*.

[6] Petrova P., Raibekas A., Pevsner J. (2003). MANF: a new mesencephalic, astrocyte-derived neurotrophic factor with selectivity for dopaminergic neurons. *Journal of molecular neuroscience: MN*.

[7] Palgi M., Lindstrom R., Peranen J., Piepponen T. P., Saarma M., Heino T. I. (2009). Evidence that DmMANF is an invertebrate neurotrophic factor supporting dopaminergic neurons. *Proceedings of the National Academy of Sciences of the United States of America*.

[8] Zhang J., Cai Q., Jiang M. (2017). Mesencephalic astrocyte-derived neurotrophic factor alleviated 6-OHDA-induced cell damage via ROS-AMPK/mTOR mediated autophagic inhibition. *Experimental Gerontology*.

[9] Zhang J., Tong W., Sun H. (2017). Nrf2-mediated neuroprotection by MANF against 6-OHDA-induced cell damage via PI3K/AKT/GSK3*β* pathway. *Experimental Gerontology*.

[10] Sun H., Jiang M., Fu X. (2017). Mesencephalic astrocyte-derived neurotrophic factor reduces cell apoptosis via upregulating HSP70 in SHSY-5Y cells. *Translational Neurodegeneration*.

[11] Zhang Z., Shen Y., Luo H. (2018). MANF protects dopamine neurons and locomotion defects from a human *α*-synuclein induced Parkinson's disease model in *C. elegans* by regulating ER stress and autophagy pathways. *Experimental Neurology*.

[12] Liu Y., Zhang J., Jiang M., Cai Q., Fang J., Jin L. (2018). MANF improves the MPP^+^/MPTP-induced Parkinson's disease via improvement of mitochondrial function and inhibition of oxidative stress. *American Journal of Translational Research*.

[13] Xilouri M., Brekk O. R., Stefanis L. (2016). Autophagy and alpha-synuclein: relevance to Parkinson's disease and related synucleopathies. *Movement Disorders*.

[14] Wong Y. C., Krainc D. (2016). Lysosomal trafficking defects link Parkinson's disease with Gaucher's disease. *Movement Disorders*.

[15] Bourdenx M., Martin-Segura A., Scrivo A. (2021). Chaperone-mediated autophagy prevents collapse of the neuronal metastable proteome. *Cell*.

[16] Yu L., Chen Y., Tooze S. A. (2018). Autophagy pathway: cellular and molecular mechanisms. *Autophagy*.

[17] Palgi M., Greco D., Lindstrom R., Auvinen P., Heino T. I. (2012). Gene expression analysis of Drosophilaa Manf mutants reveals perturbations in membrane traffic and major metabolic changes. *BMC Genomics*.

[18] Pajares M., Rojo A. I., Arias E., Díaz-Carretero A., Cuervo A. M., Cuadrado A. (2018). Transcription factor NFE2L2/NRF2 modulates chaperone-mediated autophagy through the regulation of LAMP2A. *Autophagy*.

[19] Wang Y., Zhang J., Huang Z. H. (2017). Isodeoxyelephantopin induces protective autophagy in lung cancer cells via Nrf2-p62-keap1 feedback loop. *Cell Death & Disease*.

[20] Gan L., Vargas M. R., Johnson D. A., Johnson J. A. (2012). Astrocyte-specific overexpression of Nrf2 delays motor pathology and synuclein aggregation throughout the CNS in the alpha-synuclein mutant (A53T) mouse model. *The Journal of Neuroscience*.

[21] Bartolini D., Dallaglio K., Torquato P., Piroddi M., Galli F. (2018). Nrf2-p62 autophagy pathway and its response to oxidative stress in hepatocellular carcinoma. *Translational Research*.

[22] Cuervo A. M., Stefanis L., Fredenburg R., Lansbury P. T., Sulzer D. (2004). Impaired degradation of mutant alpha-synuclein by chaperone-mediated autophagy. *Science*.

[23] Brás I. C., Xylaki M., Outeiro T. F. (2020). Mechanisms of alpha-synuclein toxicity: an update and outlook. *Progress in Brain Research*.

[24] Yang C., Gao Y. (2020). Mesencephalic astrocyte-derived neurotrophic factor: a treatment option for Parkinson's disease. *Frontiers in Bioscience (Landmark edition)*.

[25] Ho P. W., Leung C. T., Liu H. (2020). Age-dependent accumulation of oligomeric SNCA/*α*-synuclein from impaired degradation in mutant LRRK2 knockin mouse model of Parkinson disease: role for therapeutic activation of chaperone-mediated autophagy (CMA). *Autophagy*.

[26] Sala G., Marinig D., Arosio A., Ferrarese C. (2016). Role of chaperone-mediated autophagy dysfunctions in the pathogenesis of Parkinson's disease. *Frontiers in Molecular Neuroscience*.

[27] Xu C. Y., Kang W. Y., Chen Y. M. (2017). DJ-1 inhibits *α*-synuclein aggregation by regulating chaperone-mediated autophagy. *Frontiers in Aging Neuroscience*.

[28] Bonam S. R., Tranchant C., Muller S. (2021). Autophagy-lysosomal pathway as potential therapeutic target in Parkinson's disease. *Cell*.

[29] Fellner L., Gabassi E., Haybaeck J., Edenhofer F. (2021). Autophagy in *α*-synucleinopathies-an overstrained system. *Cells*.

[30] Wu H., Chen S., Ammar A. B. (2015). Crosstalk between macroautophagy and chaperone-mediated autophagy: implications for the treatment of neurological diseases. *Molecular Neurobiology*.

[31] Ma Q. (2013). Role of nrf2 in oxidative stress and toxicity. *Annual Review of Pharmacology and Toxicology*.

[32] Niu Y., Zhang J., Dong M. (2021). Nrf2 as a potential target for Parkinson's disease therapy. *Journal of Molecular Medicine (Berlin, Germany)*.

[33] Zgorzynska E., Dziedzic B., Walczewska A. (2021). An overview of the Nrf2/ARE pathway and its role in neurodegenerative diseases. *International Journal of Molecular Sciences*.

[34] Jain A., Lamark T., Sjottem E. (2010). *p62/SQSTM1* is a target gene for transcription factor NRF2 and creates a positive feedback loop by inducing antioxidant response element-driven gene transcription. *The Journal of Biological Chemistry*.

[35] Jo C., Gundemir S., Pritchard S., Jin Y. N., Rahman I., Johnson G. V. (2014). Nrf2 reduces levels of phosphorylated tau protein by inducing autophagy adaptor protein NDP52. *Nature Communications*.

[36] Pajares M., Jimenez-Moreno N., Garcia-Yague A. J. (2016). Transcription factor NFE2L2/NRF2 is a regulator of macroautophagy genes. *Autophagy*.

[37] Ji C. H., Kwon Y. T. (2017). Crosstalk and interplay between the ubiquitin-proteasome system and autophagy. *Molecules and Cells*.

[38] Zhao Z., Li F., Ning J. (2021). Novel compound FLZ alleviates rotenone-induced PD mouse model by suppressing TLR4/MyD88/NF- *κ* B pathway through microbiota-gut-brain axis. *Acta Pharmaceutica Sinica B*.

[39] Liu J., Liu W., Lu Y. (2018). Piperlongumine restores the balance of autophagy and apoptosis by increasing BCL2 phosphorylation in rotenone-induced Parkinson disease models. *Autophagy*.

[40] Crompe B., Aristieta A., Leblois A., Elsherbiny S., Boraud T., Mallet N. P. (2020). The globus pallidus orchestrates abnormal network dynamics in a model of Parkinsonism. *Nature Communications*.

[41] Tai C. H., Pan M. K., Lin J. J., Huang C. S., Yang Y. C., Kuo C. C. (2012). Subthalamic discharges as a causal determinant of Parkinsonian motor deficits. *Annals of Neurology*.

