# Supplementary figures and images for: MANF Inhibits α-Synuclein Accumulation through Activation of Autophagic Pathways
Source: Oxid Med Cell Longev. 2022 Jul 8;2022:7925686. doi: 10.1155/2022/7925686 (PMC9286947; doi:10.1155/2022/7925686)

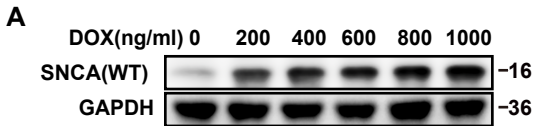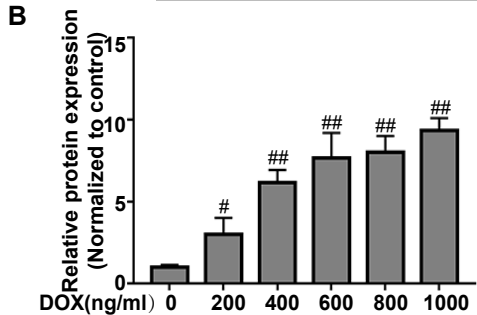

**C**

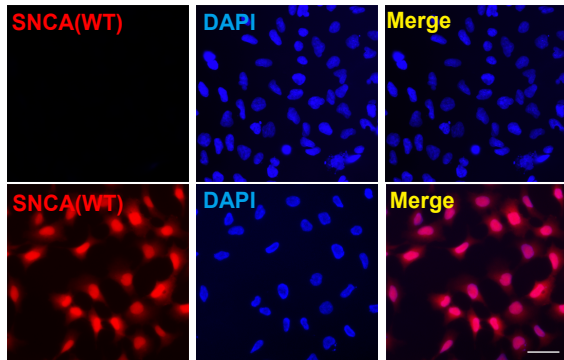

Supplement: Supplementary Materials — Figure S1: western blot analysis and immunofluorescence staining of DOX-induced expression of WT SNCA. Figure S2: western blot and immunofluorescence analysis of DOX-induced expression of A53T SNCA. Figure S3: cell viability analysis of SNCAWT SH-SY5Y cells after MANF treatment. [file 7925686.f1.zip › FigS1.pdf]

**A**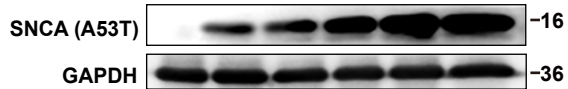**B**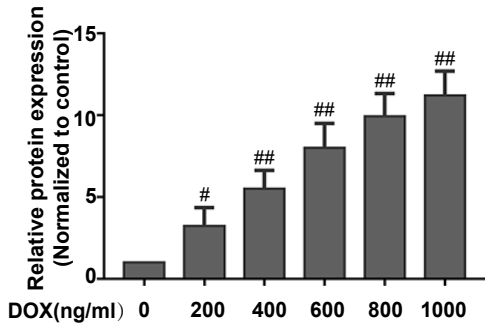**C**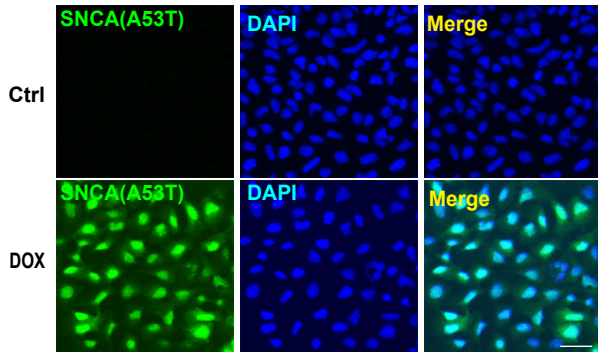

Supplement: Supplementary Materials — Figure S1: western blot analysis and immunofluorescence staining of DOX-induced expression of WT SNCA. Figure S2: western blot and immunofluorescence analysis of DOX-induced expression of A53T SNCA. Figure S3: cell viability analysis of SNCAWT SH-SY5Y cells after MANF treatment. [file 7925686.f1.zip › FigS2.pdf]

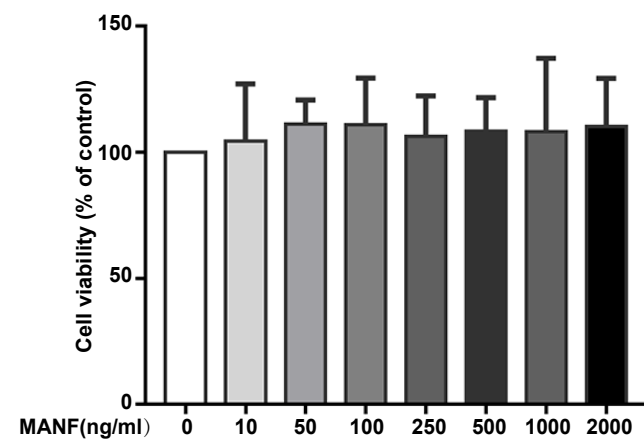

Supplement: Supplementary Materials — Figure S1: western blot analysis and immunofluorescence staining of DOX-induced expression of WT SNCA. Figure S2: western blot and immunofluorescence analysis of DOX-induced expression of A53T SNCA. Figure S3: cell viability analysis of SNCAWT SH-SY5Y cells after MANF treatment. [file 7925686.f1.zip › FigS3.pdf]
